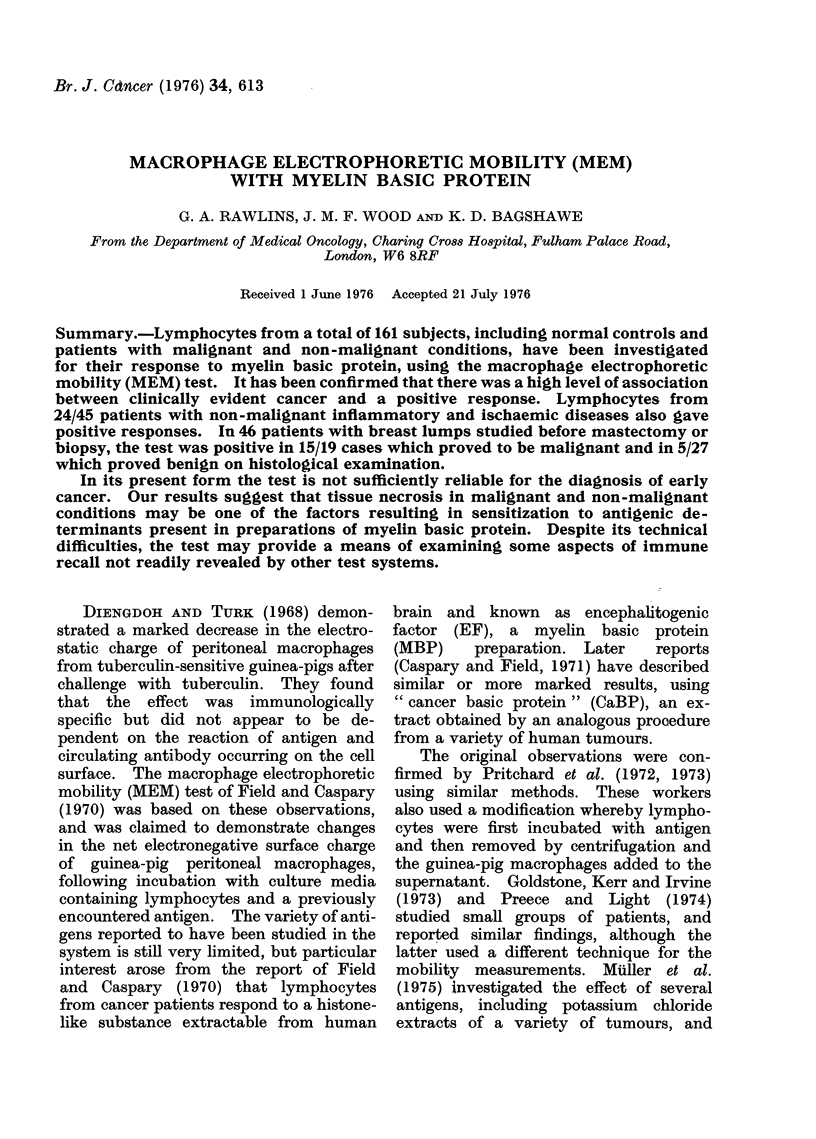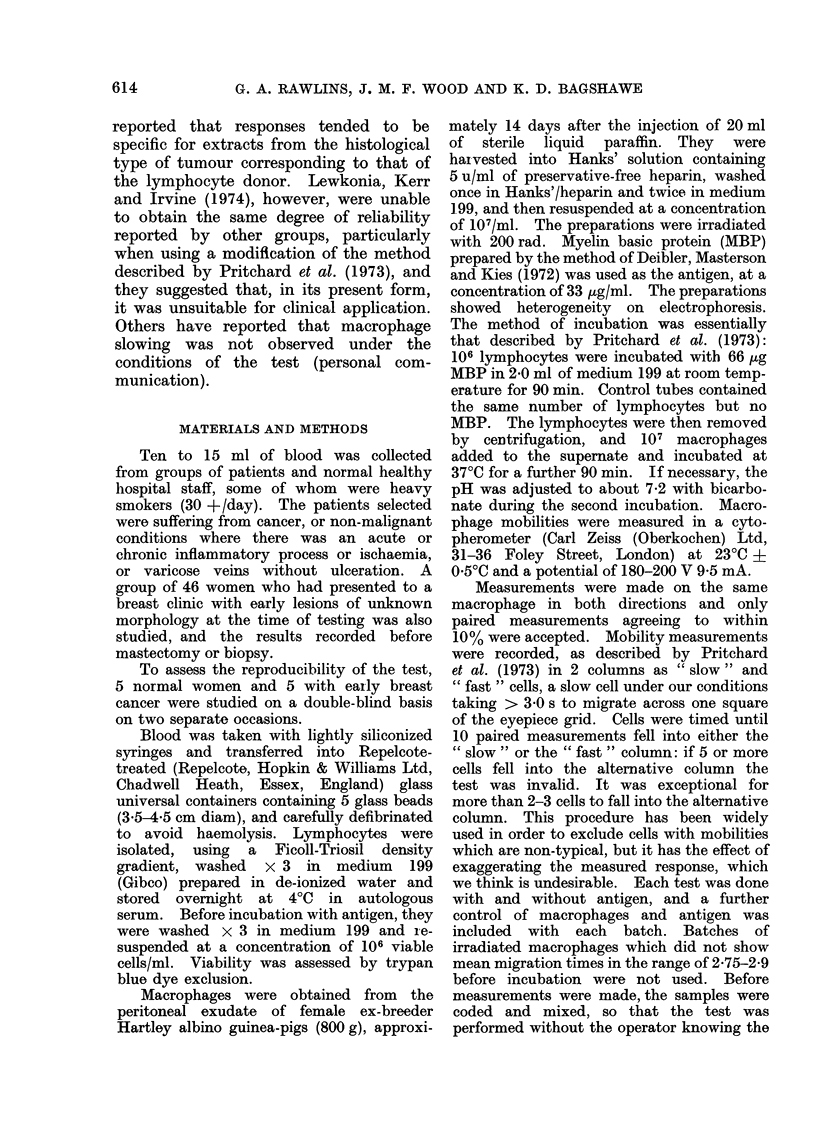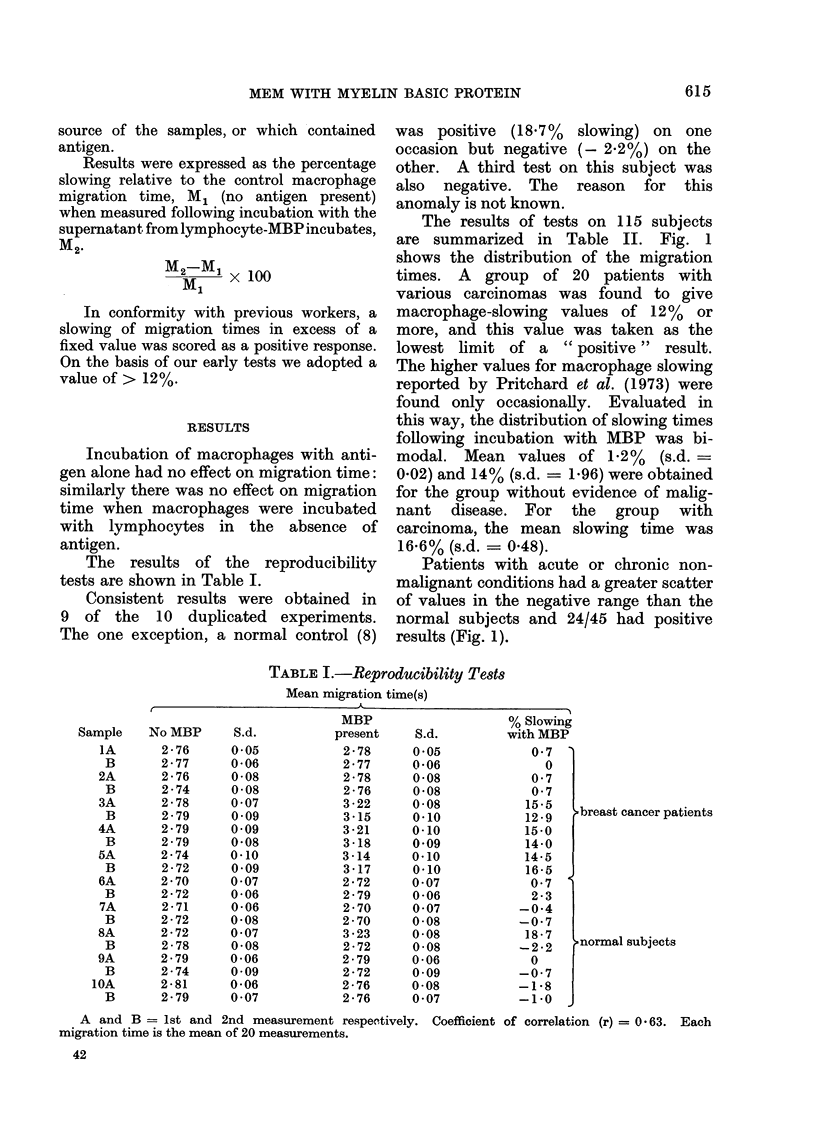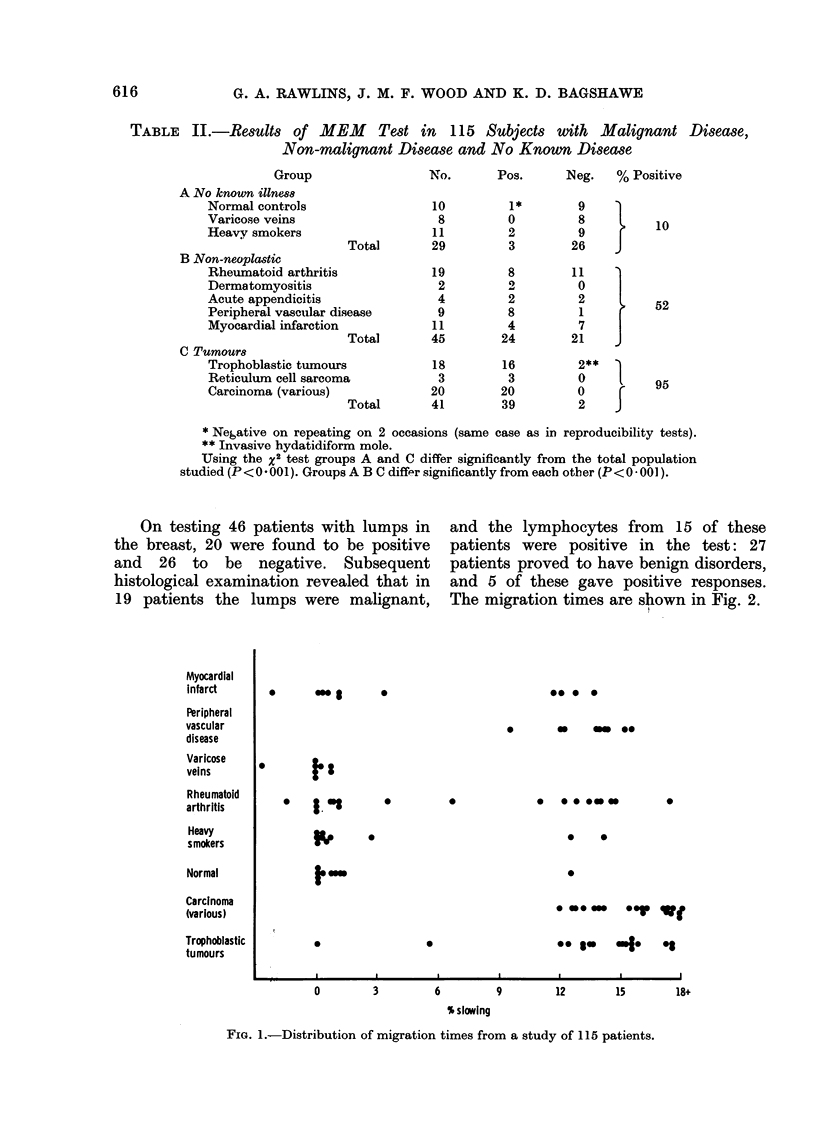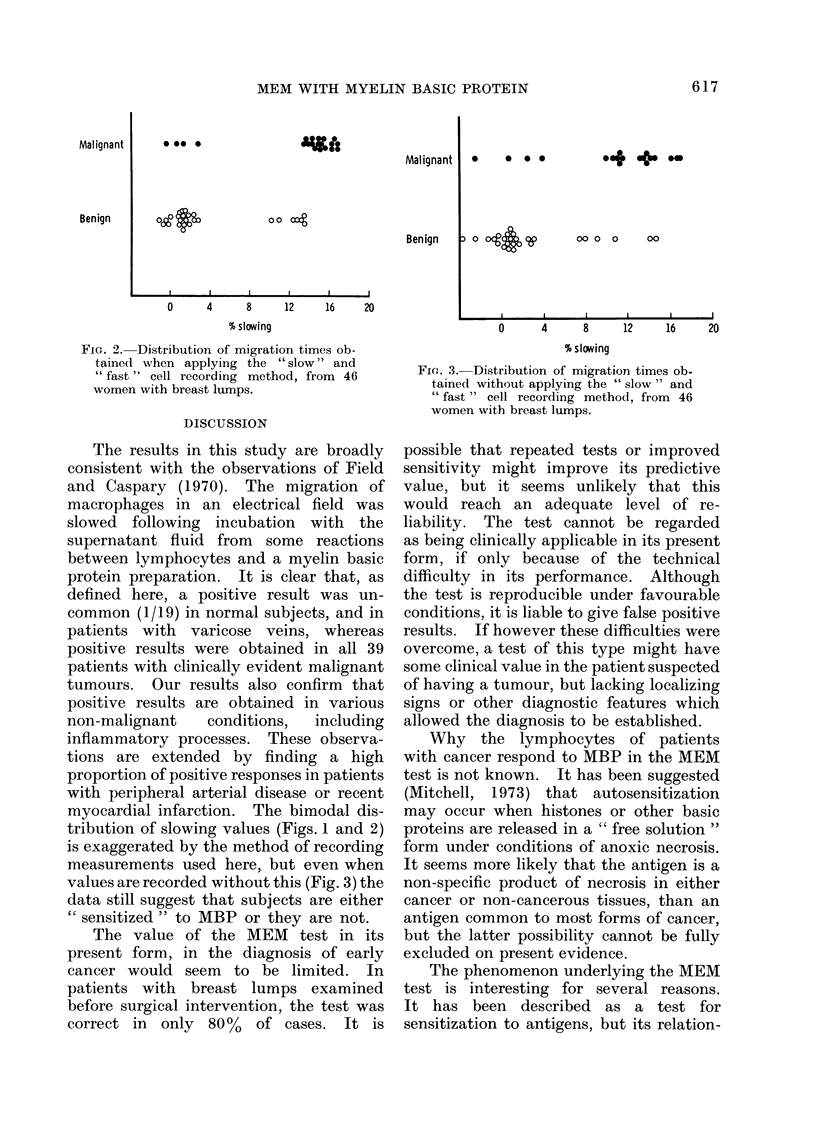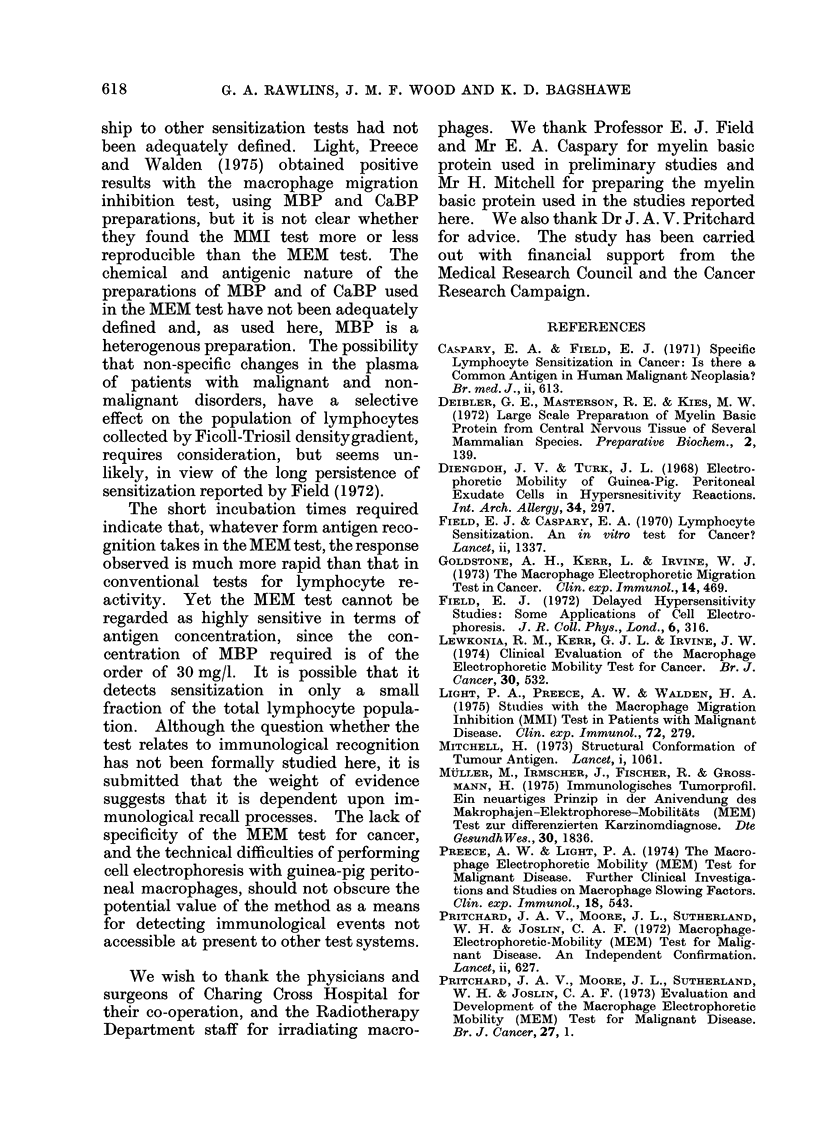# Macrophage electrophoretic mobility (MEM) with myelin basic protein.

**DOI:** 10.1038/bjc.1976.221

**Published:** 1976-12

**Authors:** G. A. Rawlins, J. M. Wood, K. D. Bagshawe

## Abstract

Lymphocytes from a total of 161 subjects, including normal controls and patients with malignant and non-malignant conditions, have been investigated for their response to myelin basic protein, using the macrophage electrophoretic mobility (MEM) test. It has been confirmed that there was a high level of association between clinically evident cancer and a positive response. Lymphocytes from 24/25 patients with non-malignant inflammatory and ischaemic diseases also gave positive responses. In 46 patients with breast lumps studied before mastectomy or biopsy, the test was positive in 15/19 cases which proved to be malignant and in 5/27 which proved benign on histological examination. In its present form the test is not sufficiently reliable for the diagnosis of early cancer. Our results suggest that tissue necrosis in malignant and non-malignant conditions may be one of the factors resulting in sensitization to antigenic determinants present in preparations of myelin basic protein. Despite its technical difficulties, the test may provide a means of examing some aspects of immune recall not readily revealed by other test systems.


Br. J. Cancer (1976) 34, 613

### MACROPHAGE ELECTROPHORETIC MOBILITY (MEM) WITH MYELIN BASIC PROTEIN

G. A. RAWLINS, J. M. F. WOOD AND K. D. BAGSHAWE

*From the Department of Medical Oncology, Charing Cross Hospital, Fulham Palace Road, London, W6 8RF*

Received 1 June 1976 Accepted 21 July 1976

**Summary.—Lymphocytes from a total of 161 subjects, including normal controls and patients with malignant and non-malignant conditions, have been investigated for their response to myelin basic protein, using the macrophage electrophoretic mobility (MEM) test. It has been confirmed that there was a high level of association between clinically evident cancer and a positive response. Lymphocytes from 24/45 patients with non-malignant inflammatory and ischaemic diseases also gave positive responses. In 46 patients with breast lumps studied before mastectomy or biopsy, the test was positive in 15/19 cases which proved to be malignant and in 5/27 which proved benign on histological examination.**

**In its present form the test is not sufficiently reliable for the diagnosis of early cancer. Our results suggest that tissue necrosis in malignant and non-malignant conditions may be one of the factors resulting in sensitization to antigenic determinants present in preparations of myelin basic protein. Despite its technical difficulties, the test may provide a means of examining some aspects of immune recall not readily revealed by other test systems.**

DIENGDOH AND TURK (1968) demonstrated a marked decrease in the electrostatic charge of peritoneal macrophages from tuberculin-sensitive guinea-pigs after challenge with tuberculin. They found that the effect was immunologically specific but did not appear to be dependent on the reaction of antigen and circulating antibody occurring on the cell surface. The macrophage electrophoretic mobility (MEM) test of Field and Caspary (1970) was based on these observations, and was claimed to demonstrate changes in the net electronegative surface charge of guinea-pig peritoneal macrophages, following incubation with culture media containing lymphocytes and a previously encountered antigen. The variety of antigens reported to have been studied in the system is still very limited, but particular interest arose from the report of Field and Caspary (1970) that lymphocytes from cancer patients respond to a histone-like substance extractable from human brain and known as encephalitogenic factor (EF), a myelin basic protein (MBP) preparation. Later reports (Caspary and Field, 1971) have described similar or more marked results, using " cancer basic protein " (CaBP), an extract obtained by an analogous procedure from a variety of human tumours.

The original observations were confirmed by Pritchard *et al.* (1972, 1973) using similar methods. These workers also used a modification whereby lymphocytes were first incubated with antigen and then removed by centrifugation and the guinea-pig macrophages added to the supernatant. Goldstone, Kerr and Irvine (1973) and Preece and Light (1974) studied small groups of patients, and reported similar findings, although the latter used a different technique for the mobility measurements. Müller *et al.* (1975) investigated the effect of several antigens, including potassium chloride extracts of a variety of tumours, and reported that responses tended to be specific for extracts from the histological type of tumour corresponding to that of the lymphocyte donor. Lewkonia, Kerr and Irvine (1974), however, were unable to obtain the same degree of reliability reported by other groups, particularly when using a modification of the method described by Pritchard *et al.* (1973), and they suggested that, in its present form, it was unsuitable for clinical application. Others have reported that macrophage slowing was not observed under the conditions of the test (personal communication).

### MATERIALS AND METHODS

Ten to 15 ml of blood was collected from groups of patients and normal healthy hospital staff, some of whom were heavy smokers (30 +/day). The patients selected were suffering from cancer, or non-malignant conditions where there was an acute or chronic inflammatory process or ischaemia, or varicose veins without ulceration. A group of 46 women who had presented to a breast clinic with early lesions of unknown morphology at the time of testing was also studied, and the results recorded before mastectomy or biopsy.

To assess the reproducibility of the test, 5 normal women and 5 with early breast cancer were studied on a double-blind basis on two separate occasions.

Blood was taken with lightly siliconized syringes and transferred into Repelcote-treated (Repelcote, Hopkin & Williams Ltd, Chadwell Heath, Essex, England) glass universal containers containing 5 glass beads (3·5–4·5 cm diam), and carefully defibrinated to avoid haemolysis. Lymphocytes were isolated, using a Ficoll-Triosil density gradient, washed × 3 in medium 199 (Gibco) prepared in de-ionized water and stored overnight at 4°C in autologous serum. Before incubation with antigen, they were washed × 3 in medium 199 and resuspended at a concentration of $10^6$ viable cells/ml. Viability was assessed by trypan blue dye exclusion.

Macrophages were obtained from the peritoneal exudate of female ex-breeder Hartley albino guinea-pigs (800 g), approximately 14 days after the injection of 20 ml of sterile liquid paraffin. They were harvested into Hanks' solution containing 5 u/ml of preservative-free heparin, washed once in Hanks'/heparin and twice in medium 199, and then resuspended at a concentration of $10^7$/ml. The preparations were irradiated with 200 rad. Myelin basic protein (MBP) prepared by the method of Deibler, Masterson and Kies (1972) was used as the antigen, at a concentration of 33 $\mu$g/ml. The preparations showed heterogeneity on electrophoresis. The method of incubation was essentially that described by Pritchard *et al.* (1973): $10^6$ lymphocytes were incubated with 66 $\mu$g MBP in 2·0 ml of medium 199 at room temperature for 90 min. Control tubes contained the same number of lymphocytes but no MBP. The lymphocytes were then removed by centrifugation, and $10^7$ macrophages added to the supernate and incubated at 37°C for a further 90 min. If necessary, the pH was adjusted to about 7·2 with bicarbonate during the second incubation. Macrophage mobilities were measured in a cytopherometer (Carl Zeiss (Oberkochen) Ltd, 31–36 Foley Street, London) at 23°C ± 0·5°C and a potential of 180–200 V 9·5 mA.

Measurements were made on the same macrophage in both directions and only paired measurements agreeing to within 10% were accepted. Mobility measurements were recorded, as described by Pritchard *et al.* (1973) in 2 columns as " slow " and " fast " cells, a slow cell under our conditions taking > 3·0 s to migrate across one square of the eyepiece grid. Cells were timed until 10 paired measurements fell into either the " slow " or the " fast " column: if 5 or more cells fell into the alternative column the test was invalid. It was exceptional for more than 2–3 cells to fall into the alternative column. This procedure has been widely used in order to exclude cells with mobilities which are non-typical, but it has the effect of exaggerating the measured response, which we think is undesirable. Each test was done with and without antigen, and a further control of macrophages and antigen was included with each batch. Batches of irradiated macrophages which did not show mean migration times in the range of 2·75–2·9 before incubation were not used. Before measurements were made, the samples were coded and mixed, so that the test was performed without the operator knowing the source of the samples, or which contained antigen.

Results were expressed as the percentage slowing relative to the control macrophage migration time, $M_1$ (no antigen present) when measured following incubation with the supernatant from lymphocyte-MBP incubates, $M_2$.

$$\frac{M_2-M_1}{M_1} \times 100$$

In conformity with previous workers, a slowing of migration times in excess of a fixed value was scored as a positive response. On the basis of our early tests we adopted a value of > 12%.

### RESULTS

Incubation of macrophages with antigen alone had no effect on migration time: similarly there was no effect on migration time when macrophages were incubated with lymphocytes in the absence of antigen.

The results of the reproducibility tests are shown in Table I.

Consistent results were obtained in 9 of the 10 duplicated experiments. The one exception, a normal control (8) was positive (18·7% slowing) on one occasion but negative (− 2·2%) on the other. A third test on this subject was also negative. The reason for this anomaly is not known.

The results of tests on 115 subjects are summarized in Table II. Fig. 1 shows the distribution of the migration times. A group of 20 patients with various carcinomas was found to give macrophage-slowing values of 12% or more, and this value was taken as the lowest limit of a "positive" result. The higher values for macrophage slowing reported by Pritchard *et al.* (1973) were found only occasionally. Evaluated in this way, the distribution of slowing times following incubation with MBP was bimodal. Mean values of 1·2% (s.d. = 0·02) and 14% (s.d. = 1·96) were obtained for the group without evidence of malignant disease. For the group with carcinoma, the mean slowing time was 16·6% (s.d. = 0·48).

Patients with acute or chronic nonmalignant conditions had a greater scatter of values in the negative range than the normal subjects and 24/45 had positive results (Fig. 1).

TABLE I.—*Reproducibility Tests*

| Sample | Mean migration time(s): No MBP | S.d. | MBP present | S.d. | % Slowing with MBP | |
|---|---|---|---|---|---|---|
| 1A | 2·76 | 0·05 | 2·78 | 0·05 | 0·7 | breast cancer patients |
| B | 2·77 | 0·06 | 2·77 | 0·06 | 0 | |
| 2A | 2·76 | 0·08 | 2·78 | 0·08 | 0·7 | |
| B | 2·74 | 0·08 | 2·76 | 0·08 | 0·7 | |
| 3A | 2·78 | 0·07 | 3·22 | 0·08 | 15·5 | |
| B | 2·79 | 0·09 | 3·15 | 0·10 | 12·9 | |
| 4A | 2·79 | 0·09 | 3·21 | 0·10 | 15·0 | |
| B | 2·79 | 0·08 | 3·18 | 0·09 | 14·0 | |
| 5A | 2·74 | 0·10 | 3·14 | 0·10 | 14·5 | |
| B | 2·72 | 0·09 | 3·17 | 0·10 | 16·5 | |
| 6A | 2·70 | 0·07 | 2·72 | 0·07 | 0·7 | normal subjects |
| B | 2·72 | 0·06 | 2·79 | 0·06 | 2·3 | |
| 7A | 2·71 | 0·06 | 2·70 | 0·07 | −0·4 | |
| B | 2·72 | 0·08 | 2·70 | 0·08 | −0·7 | |
| 8A | 2·72 | 0·07 | 3·23 | 0·08 | 18·7 | |
| B | 2·78 | 0·08 | 2·72 | 0·08 | −2·2 | |
| 9A | 2·79 | 0·06 | 2·79 | 0·06 | 0 | |
| B | 2·74 | 0·09 | 2·72 | 0·09 | −0·7 | |
| 10A | 2·81 | 0·06 | 2·76 | 0·08 | −1·8 | |
| B | 2·79 | 0·07 | 2·76 | 0·07 | −1·0 | |

A and B = 1st and 2nd measurement respectively. Coefficient of correlation (r) = 0·63. Each migration time is the mean of 20 measurements.

TABLE II.—*Results of MEM Test in 115 Subjects with Malignant Disease, Non-malignant Disease and No Known Disease*

| Group | No. | Pos. | Neg. | % Positive |
|---|---|---|---|---|
| A *No known illness* | | | | |
| Normal controls | 10 | 1* | 9 | |
| Varicose veins | 8 | 0 | 8 | 10 |
| Heavy smokers | 11 | 2 | 9 | |
| Total | 29 | 3 | 26 | |
| B *Non-neoplastic* | | | | |
| Rheumatoid arthritis | 19 | 8 | 11 | |
| Dermatomyositis | 2 | 2 | 0 | |
| Acute appendicitis | 4 | 2 | 2 | 52 |
| Peripheral vascular disease | 9 | 8 | 1 | |
| Myocardial infarction | 11 | 4 | 7 | |
| Total | 45 | 24 | 21 | |
| C *Tumours* | | | | |
| Trophoblastic tumours | 18 | 16 | 2** | |
| Reticulum cell sarcoma | 3 | 3 | 0 | 95 |
| Carcinoma (various) | 20 | 20 | 0 | |
| Total | 41 | 39 | 2 | |

* Negative on repeating on 2 occasions (same case as in reproducibility tests).
** Invasive hydatidiform mole.
Using the $\chi^2$ test groups A and C differ significantly from the total population studied ($P<0\cdot001$). Groups A B C differ significantly from each other ($P<0\cdot001$).

On testing 46 patients with lumps in the breast, 20 were found to be positive and 26 to be negative. Subsequent histological examination revealed that in 19 patients the lumps were malignant, and the lymphocytes from 15 of these patients were positive in the test: 27 patients proved to have benign disorders, and 5 of these gave positive responses. The migration times are shown in Fig. 2.

FIG. 1.—Distribution of migration times from a study of 115 patients.

FIG. 2.—Distribution of migration times obtained when applying the "slow" and "fast" cell recording method, from 46 women with breast lumps.

FIG. 3.—Distribution of migration times obtained without applying the "slow" and "fast" cell recording method, from 46 women with breast lumps.

### DISCUSSION

The results in this study are broadly consistent with the observations of Field and Caspary (1970). The migration of macrophages in an electrical field was slowed following incubation with the supernatant fluid from some reactions between lymphocytes and a myelin basic protein preparation. It is clear that, as defined here, a positive result was uncommon (1/19) in normal subjects, and in patients with varicose veins, whereas positive results were obtained in all 39 patients with clinically evident malignant tumours. Our results also confirm that positive results are obtained in various non-malignant conditions, including inflammatory processes. These observations are extended by finding a high proportion of positive responses in patients with peripheral arterial disease or recent myocardial infarction. The bimodal distribution of slowing values (Figs. 1 and 2) is exaggerated by the method of recording measurements used here, but even when values are recorded without this (Fig. 3) the data still suggest that subjects are either "sensitized" to MBP or they are not.

The value of the MEM test in its present form, in the diagnosis of early cancer would seem to be limited. In patients with breast lumps examined before surgical intervention, the test was correct in only 80% of cases. It is possible that repeated tests or improved sensitivity might improve its predictive value, but it seems unlikely that this would reach an adequate level of reliability. The test cannot be regarded as being clinically applicable in its present form, if only because of the technical difficulty in its performance. Although the test is reproducible under favourable conditions, it is liable to give false positive results. If however these difficulties were overcome, a test of this type might have some clinical value in the patient suspected of having a tumour, but lacking localizing signs or other diagnostic features which allowed the diagnosis to be established.

Why the lymphocytes of patients with cancer respond to MBP in the MEM test is not known. It has been suggested (Mitchell, 1973) that autosensitization may occur when histones or other basic proteins are released in a "free solution" form under conditions of anoxic necrosis. It seems more likely that the antigen is a non-specific product of necrosis in either cancer or non-cancerous tissues, than an antigen common to most forms of cancer, but the latter possibility cannot be fully excluded on present evidence.

The phenomenon underlying the MEM test is interesting for several reasons. It has been described as a test for sensitization to antigens, but its relationship to other sensitization tests had not been adequately defined. Light, Preece and Walden (1975) obtained positive results with the macrophage migration inhibition test, using MBP and CaBP preparations, but it is not clear whether they found the MMI test more or less reproducible than the MEM test. The chemical and antigenic nature of the preparations of MBP and of CaBP used in the MEM test have not been adequately defined and, as used here, MBP is a heterogenous preparation. The possibility that non-specific changes in the plasma of patients with malignant and non-malignant disorders, have a selective effect on the population of lymphocytes collected by Ficoll-Triosil density gradient, requires consideration, but seems unlikely, in view of the long persistence of sensitization reported by Field (1972).

The short incubation times required indicate that, whatever form antigen recognition takes in the MEM test, the response observed is much more rapid than that in conventional tests for lymphocyte reactivity. Yet the MEM test cannot be regarded as highly sensitive in terms of antigen concentration, since the concentration of MBP required is of the order of 30 mg/l. It is possible that it detects sensitization in only a small fraction of the total lymphocyte population. Although the question whether the test relates to immunological recognition has not been formally studied here, it is submitted that the weight of evidence suggests that it is dependent upon immunological recall processes. The lack of specificity of the MEM test for cancer, and the technical difficulties of performing cell electrophoresis with guinea-pig peritoneal macrophages, should not obscure the potential value of the method as a means for detecting immunological events not accessible at present to other test systems.

We wish to thank the physicians and surgeons of Charing Cross Hospital for their co-operation, and the Radiotherapy Department staff for irradiating macrophages. We thank Professor E. J. Field and Mr E. A. Caspary for myelin basic protein used in preliminary studies and Mr H. Mitchell for preparing the myelin basic protein used in the studies reported here. We also thank Dr J. A. V. Pritchard for advice. The study has been carried out with financial support from the Medical Research Council and the Cancer Research Campaign.

### REFERENCES

CASPARY, E. A. & FIELD, E. J. (1971) Specific Lymphocyte Sensitization in Cancer: Is there a Common Antigen in Human Malignant Neoplasia? *Br. med. J.*, ii, 613.

DEIBLER, G. E., MASTERSON, R. E. & KIES, M. W. (1972) Large Scale Preparation of Myelin Basic Protein from Central Nervous Tissue of Several Mammalian Species. *Preparative Biochem.*, **2**, 139.

DIENGDOH, J. V. & TURK, J. L. (1968) Electrophoretic Mobility of Guinea-Pig. Peritoneal Exudate Cells in Hypersnesitivity Reactions. *Int. Arch. Allergy*, **34**, 297.

FIELD, E. J. & CASPARY, E. A. (1970) Lymphocyte Sensitization. An *in vitro* test for Cancer? *Lancet*, ii, 1337.

GOLDSTONE, A. H., KERR, L. & IRVINE, W. J. (1973) The Macrophage Electrophoretic Migration Test in Cancer. *Clin. exp. Immunol.*, **14**, 469.

FIELD, E. J. (1972) Delayed Hypersensitivity Studies: Some Applications of Cell Electrophoresis. *J. R. Coll. Phys., Lond.*, **6**, 316.

LEWKONIA, R. M., KERR, G. J. L. & IRVINE, J. W. (1974) Clinical Evaluation of the Macrophage Electrophoretic Mobility Test for Cancer. *Br. J. Cancer*, **30**, 532.

LIGHT, P. A., PREECE, A. W. & WALDEN, H. A. (1975) Studies with the Macrophage Migration Inhibition (MMI) Test in Patients with Malignant Disease. *Clin. exp. Immunol.*, **72**, 279.

MITCHELL, H. (1973) Structural Conformation of Tumour Antigen. *Lancet*, i, 1061.

MÜLLER, M., IRMSCHER, J., FISCHER, R. & GROSSMANN, H. (1975) Immunologisches Tumorprofil. Ein neuartiges Prinzip in der Anivendung des Makrophajen-Elektrophorese–Mobilitäts (MEM) Test zur differenzierten Karzinomdiagnose. *Dte GesundhWes.*, **30**, 1836.

PREECE, A. W. & LIGHT, P. A. (1974) The Macrophage Electrophoretic Mobility (MEM) Test for Malignant Disease. Further Clinical Investigations and Studies on Macrophage Slowing Factors. *Clin. exp. Immunol.*, **18**, 543.

PRITCHARD, J. A. V., MOORE, J. L., SUTHERLAND, W. H. & JOSLIN, C. A. F. (1972) Macrophage-Electrophoretic-Mobility (MEM) Test for Malignant Disease. An Independent Confirmation. *Lancet*, ii, 627.

PRITCHARD, J. A. V., MOORE, J. L., SUTHERLAND, W. H. & JOSLIN, C. A. F. (1973) Evaluation and Development of the Macrophage Electrophoretic Mobility (MEM) Test for Malignant Disease. *Br. J. Cancer*, **27**, 1.